# Myriophyllum Biochar-Supported Mn/Mg Nano-Composites as Efficient Periodate Activators to Enhance Triphenyl Phosphate Removal from Wastewater

**DOI:** 10.3390/ma17051118

**Published:** 2024-02-29

**Authors:** Hanyun Xie, Runhua Chen, Yuxia Song, Yan Shen, Fengming Song, Bo He, Xiaomei Jiang, Yifan Yin, Wenming Wang

**Affiliations:** 1 College of Environmental Science and Engineering, Central South University of Forestry and Technology, Changsha 410007, China; 20221100480@csuft.edu.cn (H.X.); chen12@csuft.edu.cn (R.C.); rz21088@126.com (Y.Y.); 2 Hunan Pilot Yanghu Reclaimed Water Co., Ltd., Changsha 410208, Chinafloriasong@foxmail.com (F.S.); 18774875888@139.com (B.H.); 13350947057@163.com (X.J.)

**Keywords:** biochar, metal complex, organic phosphate, advanced oxidation, resourcization

## Abstract

Transition metals and their oxide compounds exhibit excellent chemical reactivity; however, their easy agglomeration and high cost limit their catalysis applications. In this study, an interpolation structure of a Myriophyllum verticillatum L. biochar-supported Mn/Mg composite (Mn/Mg@MV) was prepared to degrade triphenyl phosphate (TPhP) from wastewater through the activating periodate (PI) process. Interestingly, the Mn/Mg@MV composite showed strong radical self-producing capacities. The Mn/Mg@MV system degraded 93.34% TPhP (pH 5, 10 μM) within 150 min. The experimental results confirmed that the predominant role of IO3· and the auxiliary ·OH jointly contributed to the TPhP degradation. In addition, the TPhP pollutants were degraded to various intermediates and subsequent Mg mineral phase mineralization via mechanisms like interfacial processes and radical oxidation. DFT theoretical calculations further indicated that the synergy between Mn and Mg induced the charge transfer of the carbon-based surface, leading to the formation of an ·OH radical-enriched surface and enhancing the multivariate interface process of ·OH, IO_3_, and Mn(VII) to TPhP degradation, resulting in the further formation of Mg PO_4_ mineralization.

## 1. Introduction

The production application of organophosphate esters (OPEs) as an advantageous alternative to polybrominated diphenyl ethers (PBDEs) is increasing year by year [1]. Triphenyl phosphate (TPhP), as a typical type of organophosphate ester (OPE), has caused widespread concern in recent years due to its environmental persistence, cumulative nature, and residual toxicity [2]. TPhP is often detected in various environments such as air, water, and sediments due to its wide use as a flame retardant in textiles, cable insulation, plastics, and lacquers [3]. For example, the detection frequency (DF) of TPhP in seawater samples collected off the coast of China was 89.3%, and experts believe that TPhP is widely distributed in seawater and should be included in long-term monitoring [4]. TPhP has also been shown to cause neurotoxic, cytotoxic, and developmental toxicity in organisms [5]. In addition, inorganic phosphorus (IP), a degradation residue of TPhP, is a major trigger of eutrophication in water bodies. Therefore, the development of new technologies for the efficient and thorough treatment of TPhP wastewater and the simultaneous treatment of TPhP-degraded residual IP is a challenging and urgent issue [6].

In recent years, the advanced oxidation process (AOP) has been recognized as a wastewater treatment option because of its complete degradation and the fact that most of the degradation products are non-toxic [7,8,9]. Photocatalytic degradation has been chosen as the carrier in the AOP for use in organophosphorus flame retardants [10]. However, high costs and harsh activation conditions are the main bottlenecks related to its application. Another researcher considered the degradation of TPhP via the activation of persulfate using the novel nanomaterial CoFe_2_O_4_, which turned out to be unsatisfactory in acidic environments with a low pH [11]. Rui Hou et al. tried a new technique using a bioelectrochemical system for the enhancement of TPhP degradation efficiency, but it was obvious that the bio-domestication time was long, and it took a full 72 h for a 1.5 mg/L solution of TPhP to be completely degraded [12]. The study of biochar is a research hotspot due to biochar’s large surface area [13], rich pore space [14], and soil-friendly properties [15]. Wetland plants are harvested at the end of their lives, making them an infallible choice as feedstock for biochar and an environmentally friendly model [16,17]. However, biochar itself hardly produces strong oxidizing substances such as hydroxyl radicals in advanced oxidation [18], and its adsorption and desorption performances before being used as a carrier are not significant enough [19]; however, solving the above difficulties in the application of biochar is a worthwhile pursuit [20]. Therefore, attention has been turned to the innovative synthesis and application of biochar catalyst materials to solve the above problems [21,22]. Yaru Peng et al. applied biochar to the pretreatment process and significantly increased its surface area and adsorption properties [23]. In a recent study in 2023, Yunjiang Yu et al. constructed a persulfate oxidation system by preparing a novel material composite of biochar and pinnatite, which was able to remove 90% of TPhP in 480 min at a TPhP concentration of 10 μM, and proposed a possible degradation pathway for TPhP [24].

Although there has been a considerable amount of research studying the AOP system very thoroughly, the storage of liquid oxidizers and transportation safety issues have been two of the difficulties plaguing scientists [25]. In contrast, periodate (PI) (E_0_ = +1.6 V) has been elevated into the hot spot of recent research due to its better thermal stability and ease of transportation and storage [26]. More importantly, persulfate advanced oxidation systems generally struggle to perform effectively under acidic conditions [11]. PI was indeed reported to have excellent degradation efficiency under acidic conditions. This undoubtedly expands the application prospects of PI [27]. Transition metal activators, on the other hand, are the best partners for PI because of their abundant valence states and low energy requirements [28]. The transition-metal-based advanced oxidation process (transition metal AOP) refers to the oxidant in the transition metal and transition-metal-oxide-catalyst-induced excitation that generates a strong oxidation capacity of free radicals; moreover, through electron transfer, addition, substitution, chain-breaking, and other reactions, it achieves the efficient removal of organic matter. Common oxidizing agents include hydrogen peroxide (H_2_O_2_), persulfate (PS), peroxymonosulfate (PMS), bisulfite, and periodate (PI) [29]; common transition metal catalysts include Fe(II) zero-valent iron (ZVI), modified iron (e.g., Fe-metal–organic frameworks, Fe-MOFs), Mn(II)-modified Mn (Mn-loaded novel materials), and so on [30]. Transition metal materials have been widely used in environmental remediation due to their advantages of being green, cheap, and easy to obtain, and not generating secondary pollution. Song et al. reported that the Fe(II)/PS system showed a more outstanding TPHP removal efficiency than the Fe(II)/H_2_O_2_ system. It was also pointed out that in acidic and O_2_-rich environments, excess Fe(II) was easily oxidized to Fe(III), resulting in a significant decrease in the catalytic activity of Fe(II) [31]. Zong [32] et al. utilized Fe(II) to activate PI under acidic conditions, and the contaminants sulfamethoxazole (SMX), carbamazepine (CBZ), ciprofloxacin (CIP), acetylacetonate (CIP), and sulfonyltoluidine (CIP) were used to remove the pollutants from the Fe(II)/PS system. Acetaminophen (ACT), 2,4,6-trichlorophenol (2,4,6-TCP), and bisphenol A (BPA) were significantly degraded in a short period of time, with attenuation rates ranging from 88.9 to 100%. Among these, Mn metal was reported to be the most reactive toward PI [33]. However, it is obvious that the direct presence of Mn ions in water means that they are not only difficult to recover but also harmful to the environment. Moreover, biochar, as a high-quality carrier, can easily anchor Mn on its surface [34]. Dongmei Liu et al. showed that Mn-loaded biochar-activated persulfate could remove ciprofloxacin easily with low Mn ion leaching in five reproducible experiments [35]. Meanwhile, biochar can continue to be loaded with Mg metal as it has been reported to specifically capture orthophosphate [36], which is a novel perspective for the removal of orthophosphate, a residual product of degraded organophosphorus. Notably, the introduction of active MgO may show a high adsorption capacity and selectivity for phosphate, and enhance the limited heavy metal stabilization capacities of biochar. The literature indicates that Mn stabilization mechanisms are the formation of stable Mn species, MgMn_2_O_4_, MnO(OH)_2_, etc., through direct and indirect interactions with MgO. The main component of orthophosphoric acid captured by Mg-related groups is magnesium phosphate, which is one of the main components of phosphate fertilizers [37]. Combined with the soil affinity of biochar, after the treatment of orthophosphate, biochar can be biologically utilized through the desorption process, fulfilling the function of a biochar soil conditioner [38]. This phosphorus release method is a slow-release process, which is more friendly to the environment compared with the direct application of phosphorus fertilizer [39].

Herein, we first present a novel combined process for the MnxOy chemical co-precipitation method and MgO-crosslinking for innovative biochar synthesis. MnxOy is implemented to activate PI, while the environmentally friendly MgO can specifically bind inorganic phosphate and enhance the limited heavy metal stabilization capacities of biochar. Our results demonstrate that Mn/Mg@MV could significantly improve the TPhP removal rate while effectively removing inorganic phosphorus. This study was carried out as follows: (1) a one-step synthesis of Mn/Mg site-based BC (Mn/Mg@MV) with excellent PI activation performance was achieved without the need for additional chemicals or modification procedures; (2) the degradation effect and pathway of TPhP as a representative of the organic phosphate in the Mn/Mg@MV-PI system were comprehensively evaluated; and (3) the main catalytic active sites and RIS/ROS production mechanisms in the Mn/Mg@MV-PI system were revealed through a combination of characterization and density functional theory (DFT) calculations. This paper contributes to the advancement of the targeted conversion of BC into high-value-added carbon-based catalysts and provides novel insights into the mechanisms underlying enhanced RIS generation in PI-AOPs.

## 2. Materials and Methods

### 2.1. Chemicals and Materials

Myriophyllum verticillatum (MV) was collected from Yanghu Wetland Park, Changsha, China, and after collection, it was washed several times with deionized water, dried naturally in the sun, and then dried in an oven at 60 °C for 24 h. The dried Myriophyllum verticillatum was then made into powder using a pulverizer and passed through a 70-mesh sieve. Triphenyl phosphate (TPhP) and sodium periodate (PI) were purchased from Shanghai McLean Biochemical Co. Manganese acetate, potassium permanganate, magnesium chloride, potassium dihydrogen phosphate, potassium persulfate, ascorbic acid, ammonium molybdate, and potassium antimony tartrate were purchased from Shanghai Sinopharm Chemical Reagent Co. Methanol, hydrochloric acid and sodium hydroxide were purchased from Tianjin Hengxing Chemical Reagent Manufacturing Co. HPLC-grade acetonitrile was purchased from Shanghai Aladdin Biochemical Technology Co. All chemicals were analytically pure, and ultrapure water (≥18.25 MΩ·cm) was prepared using an ultrapure water machine at the College of Environmental Science and Engineering, Central South University of Forestry Science and Technology.

### 2.2. Synthesis of Mn/Mg@MV

An amount of 2 g of Myriophyllum verticillatum powder was stirred continuously with 100 mL of 0.15 M manganese acetate solution. Then, 100 mL of 0.1 M potassium permanganate was slowly added to the solution and stirred continuously for 30 min. The resulting sample was washed and filtered and then freeze-dried to obtain the Mn-loaded Myriophyllum verticillatum powder. The mixing and grinding of the Mn-loaded Myriophyllum verticillatum powder with MgCl_2_ was performed in a mass ratio of 1:1. Then, the powder was pyrolyzed in a tube furnace under an Ar gas atmosphere at 700 °C for 2 h with a heating rate of 10 °C/min and an Ar flow rate of 200 mL/min. Mn and Mg bimetallic-loaded biochar was obtained, which was denoted as Mn/Mg@MV. As a control, we synthesized unmodified biochar (MV) and biochar loaded only with Mg metal (Mg@MV) via pyrolysis at 700 °C for 2 h under argon gas conditions.

### 2.3. Batch Removal Experiments

The TPhP degradation capacity of Mn/Mg@MV and the removal of residual inorganic phosphorus were investigated by means of batch experiments. All TPhP degradation experiments were carried out in 150 mL brown conical flasks at 25 °C under constant temperature and darkened incubator conditions. The shaker speed was set at 180 rpm. The pH of the reaction solution was adjusted by 0.1 M HCl or NaOH. A 20 μM solution of TPhP and a dose of MV were first placed in a conical flask and mixed via thorough shaking, and then the desired stoichiometry of PI was added to initiate the reaction. At predetermined time intervals, 0.5 mL aqueous samples were taken, and the reaction was quenched by adding 0.5 mL of MeOH; this was followed by the quantitative determination of TPhP using HPLC. In addition, the optimal reaction conditions for the degradation process, such as catalyst dosage, PI concentration, and pH, were pre-evaluated. A pseudo-first-order reaction kinetic model was used to fit the experimental results. In addition, the removal of inorganic phosphorus from TPhP degradation made from several materials was evaluated by determining the TP content of the residual liquid.

### 2.4. Characterization and Analytical Methods

The residual concentration of TPhP in the batch experiments was determined using high-performance liquid chromatography (HPLC, Agilent 1200, Agilent Technologies, Santa Clara, CA, USA). The mobile phase was deionized water and acetonitrile 35:65 (V:V) at a flow rate of 1 mL/min, and the wavelength of the UV–visible spectrophotometer was 210 nm. The functional groups of the catalysts were characterized using FTIR (Thermo Scientific Nicolet iS20, Waltham, MA, USA). The surface elemental composition and chemical state of the catalysts were analyzed using XPS (Thermo Scientific K-Alpha, Boston, MA, USA).

### 2.5. DFT Calculations

The prediction of the vulnerable point positions of the contaminant TPhP in DFT calculations was performed using the Fukui function of Gaussian 16. The simulation of the electron transfer process of Mn-loaded biochar with PI, with electron differential density calculations, was performed using the CASTEP module of Material Studio 2020. Detailed parameter settings for the theoretical calculations can be found in the Supplementary Materials, Text S1.

## 3. Results and Discussion

### 3.1. Synthesis and Characterization of Mn/Mg@MV

**Figure 1:**
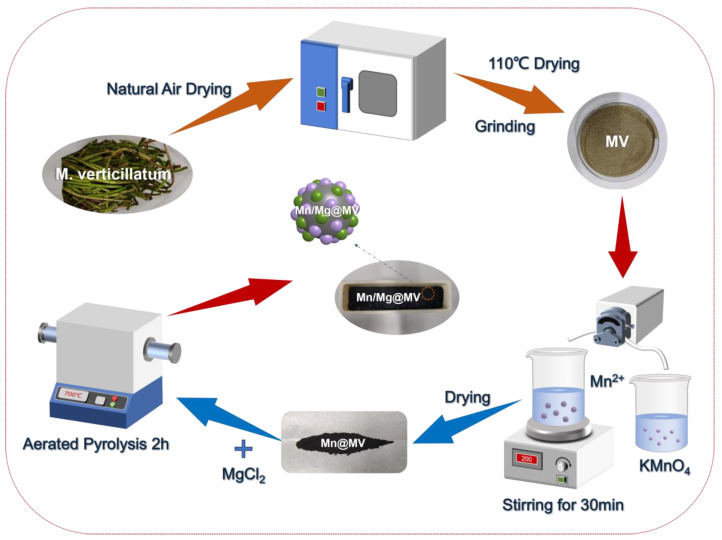
Schematic illustration of the self-assembly synthesis of Mn/Mg@MV.

The process of preparing Mn and Mg bimetallic-loaded Myriophyllum verticillatum biochar is shown in Figure 1. Briefly, the collected Myriophyllum verticillatum algae were dried and ground, and then two different valence Mn compounds were utilized to prepare MnO_2_ in a certain ratio so that the three valence states of Mn were loaded on the Myriophyllum verticillatum algae powder.

Reaction equation:

(1) $$2{{\mathrm{MnO}}_{4}}^{-}+3{\mathrm{Mn}}^{2+}+2H_{2}O\;\to {5\mathrm{MnO}}_{2}↓+4H^{+}$$

Then, the Mn-loaded Myriophyllum verticillatum powder was milled with MgCl_2_. Due to the high surface area of the biochar and its large number of hydrophilic groups, MgCl_2_ is able to separate the water in the biomass at high temperatures and generate MgO, enabling a stronger attachment to the surface of the biochar [40].

Reaction equation:

(2) $${\mathrm{MgCl}}_{2}\cdot nH_{2}O\to \mathrm{MgOHCl}+\mathrm{HCl}↑(250–450\;{}^{\circ}C)$$

(3) MgOHCl→MgO+HCl↑(450–600 °C)

Therefore,t 700 °C in a tube furnace protected by inert gas [41]. As a result, only one pyrolysis process was required to obtain the simple and efficient biochar material Mn/Mg@MV that was designed by the authors of this study, which was doped with abundant metal and functional groups. In addition, the material fully exhibits our environmentally friendly design concept, as wetland plants are harvested at the end of their lives, the trace-doped Mg is a light metal, and Mn is an essential element for plant growth [42]. Therefore, our designed biochar can be used by decomposer microorganisms to survive and reproduce, and it also demonstrates the use of soil phasing as a soil conditioner. In conclusion, our designed Mn/Mg@MV can not only realize the efficient degradation of TPhP with its metal-doped catalytic activity, but it can also eliminate the environmental hazard of degrading residual inorganic phosphorus. Meanwhile, it has the potential for application in the field of soil modification [43].

**Figure 2:**
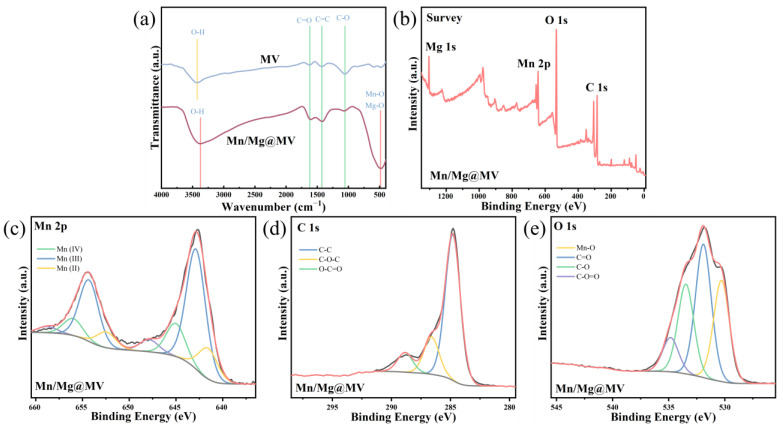
(**a**) FTIR spectra of MV and Mn/Mg@MV; (**b**) XPS survey spectra of Mn/Mg@MV; (**c**) Mn 2p; (**d**) O 1s; and (**e**) C 1s.

The surface functional groups of MV and Mn/Mg@MV were identified using Fourier-transform infrared spectroscopy (FTIR) (Figure 2). For MV, Myriophyllum verticillatum biochar without any modification, abundant functional groups were also present on the surface. The peak centered at 3430 cm^−1^ in the sample corresponds to the stretching vibration of the O-H group [44]. There are also aromatic C-O bonds (~1048 cm^−1^), aromatic C=C bonds (~1420 cm^−1^), and carbonyl C=O bonds (~1631 cm^−1^) [45]. All these organic functional groups can provide strong support to the surface modification of biochar. In addition, the surface functional groups of the modified Mn/Mg@MV composite biochar changed significantly but still retained the aromatic functional groups C=C (~1420 cm^−1^), C=O (~1631 cm^−1^), and C-O (~1048 cm^−1^) [46]. It is well known that aromatic structures act as π-electron donors and, in most cases, achieve the removal of target organic pollutants via π-π electron donor–acceptor (EDA) interactions [47] and are synergistic. On the other hand, the C-O peak intensity of Mn/Mg@MV is significantly weakened compared with that of MV, while the peak representing the metal oxide peak at Mn-O and Mg-O (482 cm^−1^) [23] is significantly enhanced, which proves the successful deposition of the metal elements Mn and Mg. This is further illustrated by the significant shift in the -OH peak [48], where Mn and Mg replaced part of the -OH and -CO functional groups and formed Mn-O and Mg-O functional groups. This suggests that Mn and Mg are not simply loaded on the surface of the biochar but are bonded through stable chemical bonds.

In order to better understand the changes in the chemical composition and surface state of Mn/Mg@MV, XPS spectroscopic studies were undertaken (Figure 2b–e). Firstly, as can be observed in the XPS survey pattern of Figure 2b, the peaks at 285.39, 532.22, 643.12, and 1304.45 eV correspond to C 1s, O 1s, Mn 2p, and Mg 2p, respectively. This result indicates that the Mn and Mg bimetals were successfully loaded on the surface of the biochar, thus ensuring that the subsequent degradation of TPhP by the material is fully carried out. Figure 2c shows the high-resolution spectral peaks of Mn 2p, with the three Mn species at 645.03, 642.86, and 641.56 eV corresponding to Mn(IV), Mn(III), and Mn(II), respectively [49]. The peak at 647.87 eV was categorized as a metallic satellite peak. Apparently, the biochar surface was uniformly doped with abundant valence states of Mn elements, which is essential for the subsequent activation of PI. The fine spectra of O 1s and C 1s are shown in Figure 2d,e. The peak at C 1s 286.6 eV and the peak at O 1s 533.47 eV prove the existence of C-O-C functional groups on the surface of the material [50], which is corroborated by the detection results of the FTIR; the peaks at C 1s 288.85 eV and O 1s 534.83 eV prove that the surface of the material is characterized by C-O=O groups [51], i.e., the presence of groups such as -COOH. While the peak at O 1s 531.91 eV proves the existence of the C=O group on the surface of the material [52], the peak at 530.29 eV proves the existence of the Mn-O bond [49]. All the above results show the presence of abundant reactive groups on the surface of the material. Interestingly, the C-O=O groups can explain the dehydration and decarboxylation reaction of biochar at high temperatures well [53], and its content is not higher than that of C-O-C groups; this laterally confirms the abundant oxygen content on the surface of Mn/Mg@MV.

### 3.2. Performance of Mn and Mg Bimetallic-Doped Myriophyllum verticillatum Biochar-Activated PI for TPhP Degradation

#### 3.2.1. TPhP Removal Performance of Different Materials

**Figure 3:**
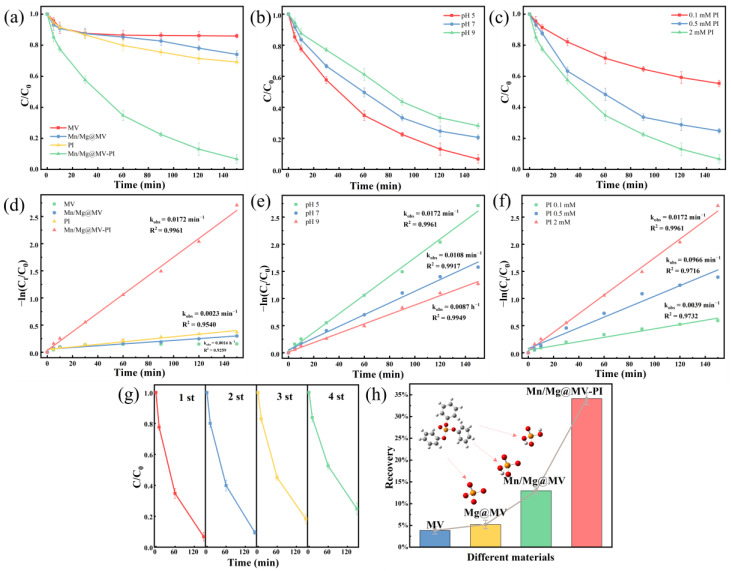
The effects of (**a**) different materials; (**b**) initial pH; (**c**) dosage of PI; (**d**–**f**) pseudo-first-order kinetics fitting results; (**g**) reusability of Mn/Mg@MV; and (**h**) inorganic phosphorus degradation residue removal. [TPhP] = 20 μM; [catalytic] = 0.75 g/L; [PI] = 2 mM; [pH] = 5; and [temperature] = 25 °C (**a**,**g**).

In order to visualize the enhancement effect of the Mn/Mg@MV-PI system on the degradation performance of TPhP, we chose unmodified biochar MV, oxidant PI alone, Mn/Mg@MV alone, and the Mn/Mg@MV-PI system for removing TPhP for a controlled evaluation. The results, as shown in Figure 3a, indicate that under the reaction condition of 150 min, Mn/Mg@MV-PI can enhance the degradation efficiency by 68.25% compared with the separate use of Mn/Mg@MV, achieving a removal efficiency of 93.34%. This result indicates that Mn/Mg@MV can effectively activate the oxidant PI to produce more oxidizing substances such as free radicals, thus degrading TPhP efficiently. In contrast, MV only removed 14.09% of TPhP under this condition. Interestingly, PI alone was able to degrade 30.80% of TPhP, which was also found to be a phenomenon in a study by Chen Yang et al. [54]. The reason for this is that PI also has some radical-producing abilities, which degrade some of the pollutants [33]. Meanwhile, pseudo-first-order reaction kinetics were used to study the removal process of TPhP via different systems [55]. It can be seen that the PI, Mn/Mg@MV, and Mn/Mg@MV-PI systems for TPhP degradation have high R^2^ values of 0.9259, 0.9540, and 0.9961, respectively. The system of TPhP removal by MV may be more suitably categorized as an adsorption reaction process. It can be seen that the k value of the system under the Mn/Mg@MV-PI system (k = 0.0172 min^−1^) is nearly 10 times higher than that of the Mn/Mg@MV (k = 0.0016 min^−1^) system. The results show that the Mn/Mg@MV-PI system could significantly enhance the treatment effect of MV on TPhP and could treat TPhP efficiently.

#### 3.2.2. Effect of Initial pH and Initial PI Dose

Solution pH may play an important role in the degradation of organic pollutants by affecting the formation of reactive oxygen species such as free radicals in the Mn/Mg@MV system. The degradation rates of TPhP after 150 min of reaction were 93.34%, 79.39%, and 71.90% at initial pH values of 5, 7, and 9, respectively. It can be clearly observed that the degradation efficiency of the Mn/Mg@MV-PI system is higher in the pH acidic environment, which was also observed by Jiahui Hu et al. [56]. Combined with the results of Kaiting Zhang et al. [57], we can presume that the reason for the increase in the degradation efficiency of TPhP due to pH elevation is theoretically supported by the low dissociation constants of the PI acid (pKa_1_ = 1.6 and pKa_2_ = 8.3) and that the main oxidant form, IO_4_^−^, is no longer dominant among the many forms of the I ions at pH > 8. From this point, the dominance of PI over persulfate in advanced oxidation systems can be seen. In addition to this, the reactivity of transition metals such as Mn is necessarily stronger under acidic conditions. Most of the transition metals generate various complexes under alkaline conditions, leading to a reduction in Mn/Mg@MV reaction sites, which significantly inhibits the electron transfer.

As seen in Figure 3c–f, the change in the PI concentration, as the main source of reactive oxygen species (ROS) in the system, is bound to have a direct impact on the total amount of ROS and the degradation rate of TPhP in the system. Therefore, the concentration of PI should be reasonably controlled to obtain the optimal degradation conditions [58]. As can be seen from Figure 3c, the PI concentration was increased from 0.1 mM to 2 mM. The degradation rate of TPhP rapidly increased from 44.67% to 93.34% within 150 min. The k obs value was increased from 0.0039 min^−1^ to 0.0172 min^−1^. Moreover, since the total amount of ROS increased with the increase in PI dosage, which increased the chance of contact with TPhP, the active site of Mn/Mg@MV itself was limited. Therefore, the TPhP degradation rate was only elevated by 18.14% when the PI dosage was increased from 0.5 mM to 2 mM.

#### 3.2.3. Evaluation of Reusability and Removal of Inorganic Phosphorus Degradation Residue

Figure 3g shows the good reusability stability of Mn/Mg@MV. After four cycles, it was still able to degrade 75.16% of TPhP within 150 min. It can be inferred that the stability of the functional group structure in the FTIR analysis and the valence transformation of Mn in the XPS analysis are the main factors for the good reuse stability of Mn/Mg@MV. On the other hand, the decrease in the catalytic performance of Mn/Mg@MV was attributed to the decrease in the adsorption capacity due to the filling of the pore structure of the biochar, the depletion of active sites, and the decrease in the specific surface area due to the incomplete activation of iodate after adsorption [59]. This was confirmed by the weakening of the peak intensity of the relevant functional groups in the FTIR analysis and the slight decrease in the metal content in the XPS analysis. In addition, because of the potential environmental hazards of inorganic phosphates in TPhP degradation residue, it is essential to consider the means of treatment applied to residual inorganic phosphates. The Mn/Mg@MV material prepared in our institute can not only realize the efficient degradation of TPhP with its metal-doped catalytic activity, but it can also eliminate the environmental hazard of degrading residual inorganic phosphorus. We compared the adsorption of orthophosphate by MV and MV@MgO prior to this study, and the results are presented in the Supplementary Materials, Figure S1. Then, we investigated the residual values of residual inorganic phosphorus degraded by TPhP under different material systems. The experiment was carried out by determining the total phosphorus values of TPhP in simulated wastewater before and after the reaction of each system and then comparing their total phosphorus removal rates. The results show that the Mn/Mg@MV-PI system contributed to a total phosphorus removal rate of 34.13%, which is 30.28% higher than the phosphorus removal rate of MV of 3.85%. This indicates that the doping of the Mg metal can effectively and specifically remove orthophosphate, which corroborates the findings of Baile Wu et al. [36]. The reason that a total phosphorus removal of only 34.13% was observed could be the incomplete nature of TPhP degradation. This low removal result is still a technical challenge in the existing studies; a large number of TPhP degradation intermediates have been detected in the degradation residue reported in current studies of TPhP degradation using novel materials. This degradation pathway phenomenon will be further discussed in the future. In conclusion, Mn/Mg@MV has excellent reusable properties and is a potential candidate for TPhP removal.

### 3.3. Mechanism Analysis

#### 3.3.1. FTIR–XPS coupling analysis before and after degradation of TPhP by Mn/Mg@MV-PI

**Figure 4:**
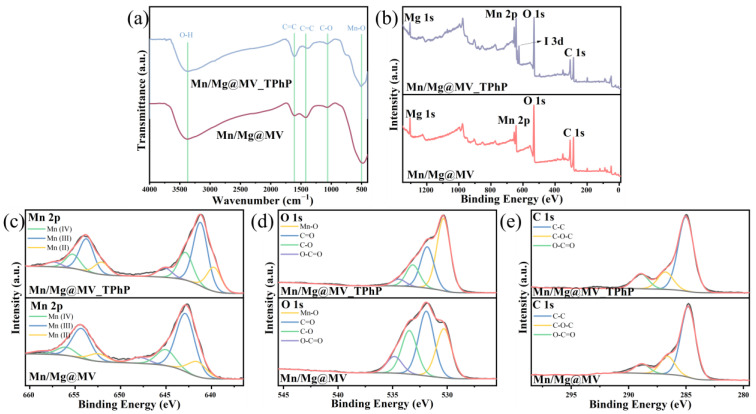
(**a**) FTIR spectra of Mn/Mg@MV_TPhP and Mn/Mg@MV; (**b**) XPS survey spectra of Mn/Mg@MV_TPhP and Mn/Mg@MV; (**c**) Mn 2p; (**d**) O 1s; and (**e**) C 1s.

To explore the changes in functional groups, FTIR analysis was performed before and after the degradation of TPhP by Mn/Mg@MV. Compared with the original samples, as shown in Figure 4a, the peak intensities of some groups of Mn/Mg@MV_TPhP used for TPhP removal changed significantly after TPhP removal. The changes in the C=C and Mn-O peak intensities suggest that the above functional groups may be involved in the reactions during TPhP removal. The increase in the C=C bond peak intensities suggests that the ligand process binds to the C=C functional groups. The weakening of the Mn-O bond peak strength may be due to the breakage of this bond, implying that the bond was consumed during the reaction and gradually weakened as the reaction progressed [60]. In particular, the bond associated with Mn-O was reduced and red-shifted, suggesting a charge transfer between the Mn-O bond and the activator [8]. However, other than this observation, the states of the other functional groups did not change significantly, indicating that the chemical structure of Mn/Mg@MV maintains some stability after use.

XPS analysis was used to further investigate the adsorption and oxidation mechanism of TPhP. When comparing the XPS survey patterns before and after the reaction, as shown in Figure 4b–e, the appearance of new peaks of I 3d can be clearly observed, and the amounts of Mn 2p and Mg 1s did not change significantly. This means that PI is indeed involved in the degradation process, and that the doped bimetallic Mn and Mg are not involved in the degradation process of TPhP via the consumption of their amounts. It is tempting to conclude that these substances have undergone some transformation to achieve both a constant flow of oxidizing substances and their reusability [61]. By analyzing the XPS fine spectra of Mn/Mg@MV before and after the degradation of TPhP, we verified our conjecture. It is obvious that the content of each valence state of Mn changed considerably during the reaction [62]. This suggests that the electron transfer process of Mn-O on Mn/Mg@MV occurs during the activation of PI; the activation of PI by Mn/Mg@MV is largely attributed to the change in the valence state of Mn. The decrease in the percentage of Mn(II) and Mn(IV) content in the Mn 2p spectra reflects their contribution to PI activation. The significant decrease in the percentage of Mn(II) may be related to the fact that Mn(II)/Mn(III) is more susceptible to redox cycling than Mn(IV)/Mn(III) [63]. The transformation of the proportion of oxygen forms in O 1s is also quite enlightening, and the increase in the percentage of Mn-O corroborates the enhancement of Mn material activity.

#### 3.3.2. Fukui Function for Susceptible Site Prediction and Electron Transfer Pathway DFT Calculations

**Figure 5:**
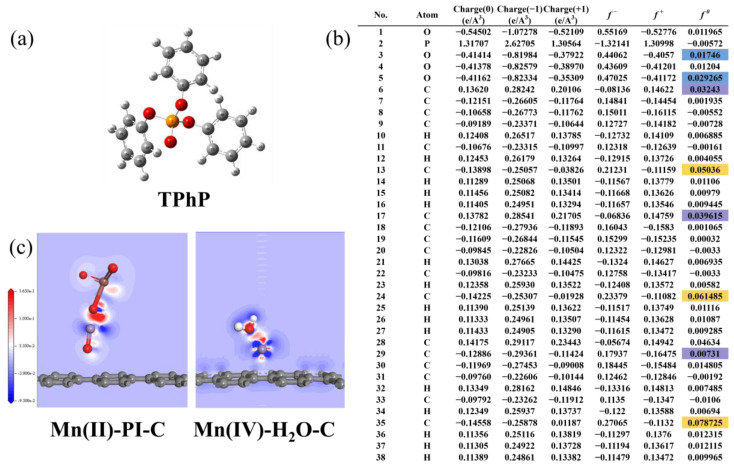
(**a**) Optimized chemical structure of TPhP; (**b**) charge distribution and Fukui index of TPhP (Fluorescent markers represent vulnerable sites); and (**c**) charge differential density modeling under the CASTEP module.

The possible attack sites on TPhP subjected to reactive oxidants were analyzed using the Fukui function. The Fukui function is a very important molecular reactivity descriptor function that can be used to predict the nature of the chemical reactions of molecules. The Fukui function can be related to three types of reactions separately (electrophilic (*f^−^*), nucleophilic (*f^+^*), and radical reactions (*f*^0^)). These three types of reactions correspond to different formulas for calculating the Fukui function, where higher values of *f*^0^ represent sites that are more susceptible to HO-radical attack. As shown in Figure 5, the calculation of the Fukui function of TPhP using DFT leads to the conclusion that C35 is the site most susceptible to HO-radical attack. This is in full agreement with the P5 pathway outlined in the study by Yunjiang Yu et al. [24]. The other relatively vulnerable site, C17, is corroborated with the P1 pathway. From this, we can also hypothesize that it is the hydroxyl radicals, etc., that are the ROS that play a major role in the reaction process in the Mn/Mg@MV-PI system, rather than the electrophilic attack participants such as non-radical species like Mn(III). This is consistent with the experimental phenomenon of the low degradation efficiency of Mn/Mg@MV using TPhP alone. These phenomena fully demonstrate the accuracy of the Fukui function in predicting the attack sites of pollutants. New insights are provided for future research directions.

FTIR–XPS studies of the active sites provide compelling evidence that the excellent catalytic performance of Mn/Mg@MV can be attributed to a range of surface structures, including oxygen functional groups and the valence transformation of Mn, although previous studies have elucidated the influential role played by oxygen-containing functional groups in PI activation [54]. However, the precise influence of the Mn-O site, the H_2_O molecular environment, on the electronic structure of biochar and the PI reaction mechanism remains elusive. Therefore, simplified models (Mn(II)-PI-C system and Mn(IV)-H_2_O-C system) were constructed to reveal the influencing mechanism of PI activation at Mn-O catalytic sites and the mechanism of Mn valence transformation. The charge density analysis performed by the CASTEP module of Material Studio demonstrated a significant electron transfer from Mn(II) to PI between the IO_4_^-^ and Mn(II) centers, which activated PI on Mn/Mg@MV to produce strong oxidizing substances such as IO_3_^−^, OH^−^, O_2_^−^, ^1^O_2_, and so on. On the other hand, Mn(IV) can be utilized again after activation. Charge density analysis shows that Mn(IV) can also undergo charge transfer through the aqueous medium in the environment, thus recovering into Mn(II) to be reused for PI activation, forming a system for the cyclic transformation of Mn valence and corroborating with the results of XPS characterization.

#### 3.3.3. Possible Mechanisms of PI Activation on Mn/Mg@MV and the Degradation Pathway of TPhP

**Figure 6:**
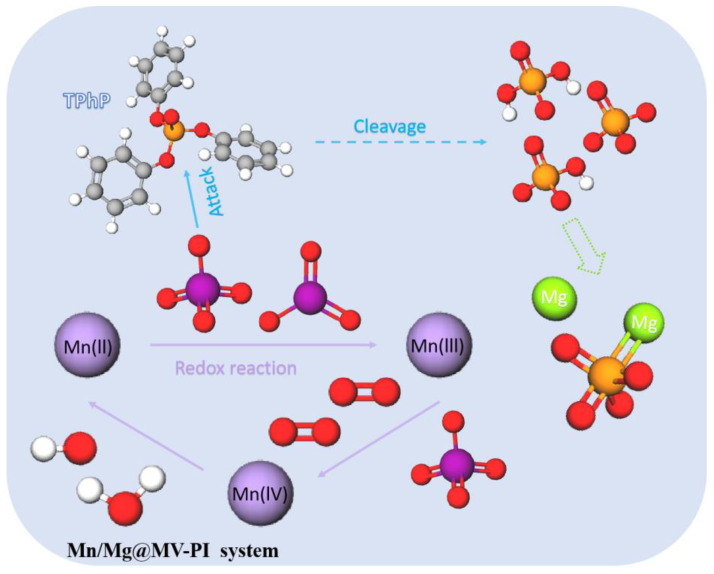
The possible removal mechanism of TPhP by Mn/Mg@MV-PI.

According to the results of laboratory batch removal experiments, as well as various characterization analyses and theoretical analyses, the degradation mechanism of TPhP in the Mn/Mg@MV-PI system is shown in Figure 6. Firstly, PI is adsorbed on the Mn(II) active site and activated to generate a large amount of IO_3_**·**, which can attack the oxygen atom sites of TPhP, while Mn(II)/Mn(III) is oxidized to higher-order Mn oxides (Mn(III)/Mn(IV)). Through a reduction in the aqueous medium, Mn is again reduced to a lower valence state, forming a closed loop of Mn valence transformation and leading to the recycling of Mn catalytic sites, while generating strong oxidizing substances such as hydroxyl radicals. On the other hand, when TPhP and its intermediates are continuously degraded, the inorganic phosphate left behind is firmly fixed by the coordination of the Mg element, so as to reduce the generation of residual inorganic phosphorus pollutants.

## 4. Conclusions

Based on the concept of sustainable development, a novel multifunctional bimetallic Myriophyllum verticillatum powder sufficiently mixed with Mn ions and Mg ions as a precursor was successfully prepared as a PI-activated catalyst via tube furnace pyrolysis in an anaerobic environment using a simple surface-loaded doping method. The Mn/Mg@MV composite possesses high-quality transition-metal-based electron-transferring properties, good PI catalytic activity, and excellent orthophosphate removal efficiency. Moreover, Mn/Mg@MV has a large specific surface area, abundant surface functional groups, carbon–carbon double bonds and carbon–oxygen bonds, and a strong electron transfer interaction with PI, resulting in excellent catalytic performance. The pseudo-first-order kinetics describe the experimental data well. In addition, Mn/Mg@MV showed a high recycling rate in at least four runs, proving its stability and reusability. Thus, this work not only demonstrates the promising practical application of Mn/Mg@MV-PI for TPhP degradation, but also opens up new directions for the development of cost-effective catalyst processes and sustainable utilization.

## Data Availability

The data presented in this study are available on request from the corresponding author.

## Supplementary Materials

The following supporting information can be downloaded at: https://www.mdpi.com/article/10.3390/ma17051118/s1, Text S1: Density functional theory (DFT) calculation analysis; Figure S1: Comparison of inorganic phosphorus adsorption on biochar before and after Mg doping; Figure S2: Adsorption properties of MV on TPhP; Figure S3: Adsorption properties of Mn/Mg@MV on TPhP; Figure S4: Double beam UV-Visible spectrophotometer for TPhP degradation; Table S1: Comparison of the properties of Mn/Mg@MV and other materials for TPhP removal [14,22,64].
