# Supplementary material for: Myriophyllum Biochar-Supported Mn/Mg Nano-Composites as Efficient Periodate Activators to Enhance Triphenyl Phosphate Removal from Wastewater
Source: Materials (Basel). 2024 Feb 29;17(5):1118. doi: 10.3390/ma17051118 (PMC10934345; doi:10.3390/ma17051118)
Supplement: Supplementary file 1 [file materials-17-01118-s001.zip › materials-2852240-supplementary.pdf]

Supplementary materials

Myriophyllum biochar supported Mn/Mg nano-composites as efficient periodate activators for enhance triphenyl phosphate removal from wastewater

Hanyun Xie[a#], Runhua Chen[a#], Yuxia Song[a*], Yan Shen[b], Fengming Song[b], Bo He[b], Xiaomei Jiang[b], Yifan Yin[a],Wenming Wang[b]*

[a]College of Environmental Science and Engineering, Central South University of Forestry and Technology, Changsha 410007, China;

[b]Hunan Pilot Yanghu Reclaimed Water Co. Ltd., Changsha 410208, China.

Corresponding author's email:

yuxiasong@csuft.edu.cn (Yuxia Song); w.m.wang@126.com (Wenming Wang).

[#]These authors contributed equally to this work.

Text S1. Density functional theory (DFT) calculation analysis

Fukui function based on the density functional theory (DFT) was employed to predict the regioselectivity of electrophilic, nucleophilic, and radical attacks towards the TPhP molecules. The building configuration optimization and single-point energy calculations was conducted by B3LYP/6-31+G(d,p) basis set, as disposed of in the Gaussian 16 software package. The atomic reactivity in TPhP was characterized by the Fukui index (f^-, f^+ and f^0) calculated as follows:

Electrophilic attack: $f_A^- = q_{N-1}^A - q_N^A$

Nucleophilic attack: $f_A^+ = q_N^A - q_{N+1}^A$

Radical attack: $f_A^0 = (q_{N-1}^A - q_{N+1}^A)/2$

Generally, the higher Fukui indexes mean more easily attacked by ROS.

Spin-polarized electronic structure calculations were performed using the package (Material Studio 2020). The valence electrons were represented with a plane wave basis set with an energy cutoff of 450 eV. Electronic exchange and correlation were described with the Perdew–Burke–Ernzerhof (PBE) functional. A 3×3 supercell of graphite (001)

surface was chosen to stabilize Mn doping and 15 Å vacuum space to avoid interactions between surface slabs. A 3×3×1 Monkhorst–Pack scheme was used to generate the k-point grid for the supercells of surface. The van der Waals (vdW) interaction was amended by the zero damping DFT-D3 method of Grimme. The convergence criteria for the self-consistent electronic structure and geometry were set to 10^{-5} eV and 0.05 eV/Å, respectively. The adsorption energy (E_{ad}) was calculated by the following equation:

$$E_{ad} = E_{total} - E_{surface} - E_{IO_4^-}$$

Where E_{total}, $E_{surface}$, and $E_{IO_4^-}$ refer to the DFT-calculated energy for total adsorbed structure, the pure surface and IO_4^-.

Figure S1. Comparison of inorganic phosphorus adsorption on biochar before and after Mg doping

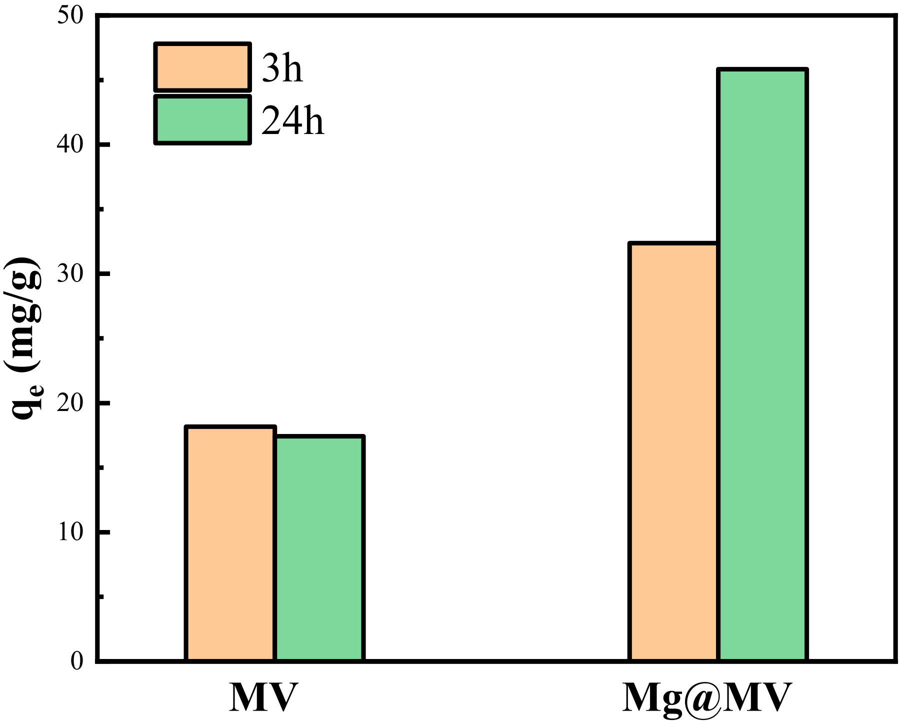

Figure S2. Adsorption properties of MV on TPhP

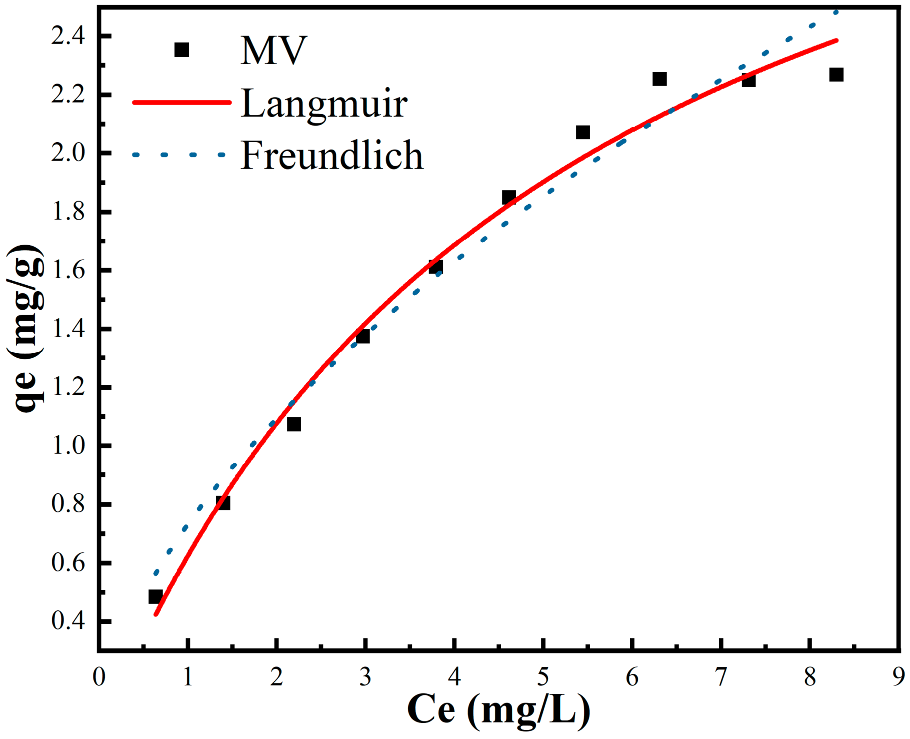

Figure S3. Adsorption properties of Mn/Mg@MV on TPhP

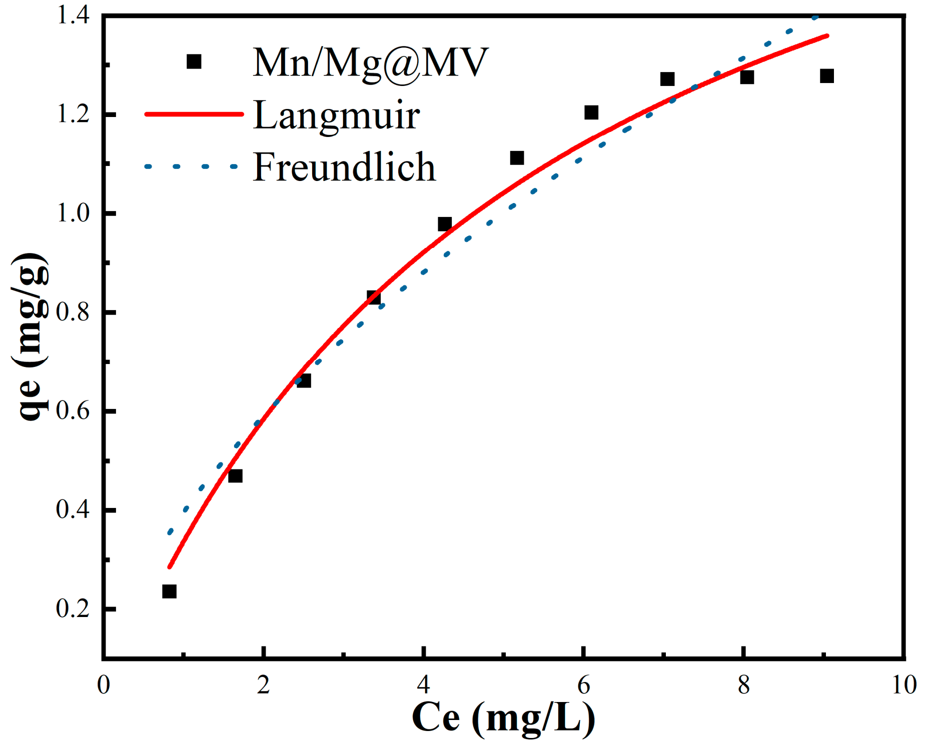

Figure S4. Double beam UV-Visible spectrophotometer for TPhP degradation

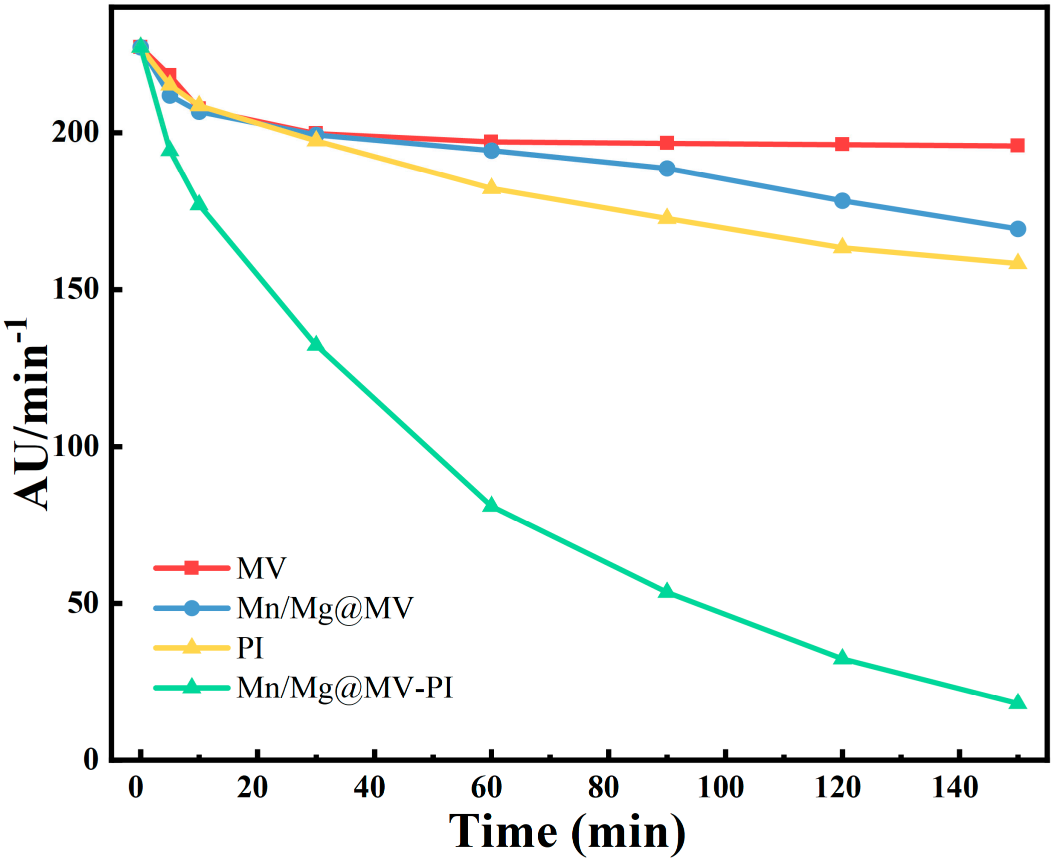

Table S1. Comparison of the properties of Mn/Mg@MV and other materials for TPhP removal.

Removal materials	Removal capacity k_{obs} (min^{-1})	References
MBC35/80@FH&PDS	**0.00723**	**[22]**
BES&Sodium acetate	**0.0009**	**[14]**
BC700@nZVI	**0.0155**	**[54]**
Mn/Mg@MV	**0.0172**	**This work**

References

[54] L. Li, J. Li, Y. Yan, R. Ma, X. Zhang, J. Wang, Y. Shen, H. Ullah, L. Lu, Removal of organophosphorus flame retardant by biochar-coated nZVI activating persulfate: Synergistic mechanism of adsorption and catalytic degradation, Environmental Pollution 331 (2023) 121880. https://doi.org/https://doi.org/10.1016/j.envpol.2023.121880.
